# Caulis Sargentodoxae Prescription Plays a Therapeutic Role with Decreased Inflammatory Cytokines in Peritoneal Fluid in the Rat Endometriosis Model

**DOI:** 10.1155/2020/9627907

**Published:** 2020-05-29

**Authors:** Mengfei Zhuang, Yang Cao, Yan Shi, Lin Yu, Yanan Niu, Tingting Zhang, Zhaogui Sun

**Affiliations:** ^1^Yueyang Hospital of Integrated Traditional Chinese and Western Medicine, Shanghai University of Traditional Chinese Medicine, Shanghai 200437, China; ^2^NHC Key Lab of Reproduction Regulation (Shanghai Institute of Planned Parenthood Research), Medical School, Fudan University, Shanghai 200032, China

## Abstract

Caulis Sargentodoxae prescription has been confirmed by the gynecological clinical observation to be effective in the treatment of endometriosis (EMs), and inflammatory cytokines were involved in EMs. In this paper, animal experiments were designed to explain anti-inflammatory mechanisms of Caulis Sargentodoxae prescription on endometriosis. The EMs model was established by autoplastic transplantation, and rats were randomly divided into seven groups: normal control group, model group, ovariectomized group, gestrinone (Western medicine) group, Caulis Sargentodoxae prescription (Chinese medicine) group, celecoxib (inhibitor) group, and combination (Chinese medicine + inhibitor) group. After oral administration for 21 days, the growth inhibitory rates of the ectopic endometria in treatment groups were evaluated, and the levels of inflammatory cytokines in the serum and peritoneal fluid were determined by ELISA, as well as the expression of prostaglandin endoperoxide synthase 2 (PTGS2) in the ectopic endometrial tissues was detected by real-time polymerase chain reaction, immunohistochemistry, and western blotting. The growth inhibitory rates of the ectopic endometria were significantly higher in the Caulis Sargentodoxae prescription group and gestrinone group, in comparison with the model group (*p* < 0.05). In the Caulis Sargentodoxae prescription group, the levels of inflammatory cytokines including IL-1, IL-2, IL-6, TNF-*α*, and PGE2 were all reduced in the serum and peritoneal fluid (*p* < 0.05). In addition, the specific expression of PTGS2 in the ectopic endometrial tissues significantly decreased in the Caulis Sargentodoxae prescription group and PTGS2 inhibitor celecoxib group both at mRNA and protein levels, but in the steroid hormone drug gestrinone group not at the mRNA level. So, Caulis Sargentodoxae prescription has a reliable therapeutic effect on the EMs by its comprehensive anti-inflammatory roles, possibly in a way different from gestrinone.

## 1. Introduction

Endometriosis (EMs) is a chronic inflammatory disease, characterized by implantation and growth of endometrial tissues outside the uterine cavity [1]. Its influences can spoil pelvic environment and immune function, damage ovarian tissues, and reduce follicular growth, leading to the occurrence of dysmenorrhea, infertility, and menstrual disorder [2]. This disabling condition is considered as one of the most frequent diseases in gynecology, affecting 15–20% of women in their reproductive age [3]. Studies have shown that 40–60% of dysmenorrhea women were found with varying degrees of EMs, and the percentage of EMs patients with infertility was thought as high as 20–30% [4]. The treatment includes surgical treatment, medication, interventional therapy, and traditional Chinese medicine treatment [5].

More studies have shown that immune regulation plays an important role in the occurrence and development of EMs. The decrease of systemic immune function is the intrinsic factor on EMs, and the change of peritoneal microenvironment to the “allowed condition” is a key for ectopic implantation and growth of the endometrial tissue [6, 7]. Excessive production of inflammatory cytokines may contribute to the further development of EMs. Interleukin (IL) is a class of cytokines involved in the activation of immune cells and the development of inflammatory responses. As an animal experimental study of Cao et al. [8] showed that when endometrial epithelia and interstitial cells were injected into the abdominal cavity of female mice for 4 hours, a large number of inflammatory cytokines such as IL-1, IL-6, and MCP-1 were produced in peritoneal fluid, which could promote adhesion and invasion of ectopic endometria.

Caulis Sargentodoxae prescription is an empirical formula of Chinese herbs created by Deying Dai, a famous expert of traditional Chinese medicine in Shanghai. After decades of clinical verification, it was found that Dai's formula had definite curative effects on EMs, which had a total clinical effective rate of ∼90% on EMs, 90.63% on menstrual fever, and 96.25% on dysmenorrhea [9–11]. It also effectively prevented endometriotic recurrence [12] after surgery and significantly improved the quality of life of EMs patients with dysmenorrhea and chronic pelvic pain [13].

On the basis of definite curative effects confirmed by clinical observation, in this research Caulis Sargentodoxae prescription was applied to inhibit the growth of ectopic endometria, and involved mechanisms were analyzed by detecting inflammatory factors with a rat model.

## 2. Materials and Methods

### 2.1. Animals

Sprague-Dawley rats for the experiments were aged 6–8 weeks, weighed 160–200 g, and from B&K Universal Group Limited Shanghai, China. They were housed under specific pathogen-free conditions in an air-conditioned room with 12 h light/12 h dark cycles and at 22–24°C with 55%–65% relative humidity. Rats had free access to food and water. All experiments were carried out in accordance with the guidelines for the Care and Use of Laboratory Animals and were approved by the Ethics Committee of Shanghai Institute of Planned Parenthood Research (SIPPR). All procedures were performed in accordance with the guidelines established by the Ethics Committee of the SIPPR.

### 2.2. Chemicals and Reagents

Caulis Sargentodoxae prescription [14] studied in this paper had been widely used in clinic, and its prescription includes Chinese traditional herbs as follows: *Sargentodoxa cuneata* (Oliv.) Rehd. et Wils., recorded in “Bencao tujing”; *Typha angustifolia* L., recorded in “Shennong bencaojing”; *Ostrea gigas* Thunberg, oyster in English, recorded in “Shennong bencaojing”; *Corydalis yanhusuo* W. T. Wang, recorded in “Leigong Paozhilun”; *Paeonia suffruticosa* Andr., recorded in “Shennong bencaojing”; *Prunus persica* Batsch, peach kernel in English, recorded in “Shennong bencaojing”; and *Cyperus rotundus* L., recorded in “Mingyi bielu”. The decoction was prepared by the pharmacy at the Yueyang Hospital of Integrated Traditional Chinese and Western Medicine, which is affiliated with the Shanghai University of Traditional Chinese Medicine, according to a conventional preparation method by boiling in water. The procedures are as follows. All herbs were soaked in cold water for about 20 minutes, boiled for 30 minutes, then the decoction was collected, and the process was repeated once again; the two decoctions were mixed, kept sterile, and refrigerated at 4°C for use. Its equivalent concentration of traditional Chinese medicine is 2.75 g/ml. The experimental dosage was determined in reference to the clinical medication by the equivalent surface area method.

The gestrinone active pharmaceutical ingredient (API) was purchased from Fengzhulin Chemexpress Co., Ltd. (Wuhan, China). The celecoxib API was purchased from Qiyi Biological Technology Co., Ltd. (Shanghai, China). Rat IL-1, IL-2, IL-6, TNF-*α*, and PGE_2_ ELISA kits were purchased from *X*-*Y* Biotechnology (Shanghai, China). The rabbit anti-PTGS2 monoclonal antibody (cat. no. 12282) was purchased from Cell Signaling Technology, Inc. (Boston, MA, USA). The rabbit anti-glyceraldehyde-3-phosphate dehydrogenase (GAPDH) polyclonal antibody (cat. no. 10494-1-AP) was purchased from Proteintech Group, Inc. (Rosemont, IL, USA).

### 2.3. Establishment of the Rat Model of EMs

According to the method of Jones [15], the EMs rat models was established under sterile conditions. The rats underwent a vertical abdominal incision after being anesthetized with 3% pelltobarbitalum natricum. A section on the left side of the uterus was removed and immediately placed into physiologic saline. The endometrium was separated from the myometrium and cut into 0.5 cm × 0.5 cm fragments. The uterine endometrial segments were sutured onto the peritoneum near blood vessels of the same rat itself. The incision was closed and disinfected. After waking up from anaesthesia, the rats were sent back to the feeding room.

### 2.4. Medicine Administration Groups and Sample Collection

At 21 days after the surgery, the rats underwent a second laparotomy to check the growth of the ectopic endometrium tissue. The volume (length × width × height) was measured with an electronic digital caliper. Rats with ectopic tissue volumes larger than 20 mm^3^ were randomly divided into groups for further experiments (Table 1). The normal control group of five normal rats without any operation was introduced as control for the model operation. To compare the different effects between Chinese medicine and Western medicine and inflammation inhibitor, we had set up seven groups: normal control group, model group, ovariectomized group (bilateral ovaries were removed), gestrinone (Western medicine) group, Caulis Sargentodoxae prescription (Chinese medicine) group, celecoxib (inhibitor) group, and combination (Chinese medicine + inhibitor) group. In the ovariectomized group, after taking a proximal midline incision at abdomen, the ovaries were pulled out by the uterus horns with tweezers, and the ovaries were cut off with ophthalmic scissors after the ovarian mesangial vessels were ligated with suture thread. Rats in seven groups received different oral administrations as listed in Table 1.

After the oral administration of medicines for 21 days, 10 ml of phosphate-buffered saline (PBS) buffer was injected into the abdominal cavity of rats after anaesthesia. Gently shook the rats for 10 min. The rat abdominal wall was punctured with a blunt needle, and the peritoneal fluid was extracted with a 10 ml syringe. Then, the abdominal wall was cut longitudinally, and blood was drawn from the abdominal aorta with a 10 ml syringe. The peritoneal fluid was extracted about 5–8 ml, and the blood was extracted about 3–5 ml. Next, the rats were sacrificed by cervical dislocation and the tissue samples were obtained by dissection. The supernatant of the peritoneal fluid and serum samples were harvested and stored at −80°C. The ectopic and eutopic endometria were collected. One half of the tissues were fixed in 4% neutral paraformaldehyde for sectioning and histological analysis, whereas the other half were frozen in liquid nitrogen for molecular detection.

### 2.5. Enzyme-Linked Immunosorbent Assay

The peritoneal fluid and serum samples were collected and stored at 2–8°C for no more than 5 days. Thereafter, the cytokine levels of IL-1, IL-2, IL-6, TNF-*α*, and PGE_2_ were measured according to the manufacturer's instructions. Standard curves were prepared, and the peritoneal fluid and serum samples were diluted in a ratio of 1 : 5 with the diluent buffer as directed by the manufacturer's instructions. Antibody incubation and washing steps were performed according to the routine protocols. After the TMB (3,3′,5,5′-Tetramethylbenzidine, TMB) color development, the absorbance at A450 was recorded on a model ELx-800 ELISA microplate reader (BIO-TEK Instruments Inc.).

### 2.6. Immuno-Histochemistry

Tissues fixed in 4% neutral paraformaldehyde were subjected to washing, dehydration, waxing, and embedding, and then cut into serial 5 *μ*m thick sections in routine. After the sections were dewaxed in xylene and rehydrated, antigen retrieval was carried out by boiling in 10 mmol/L sodium citrate buffer (pH 6.0) in a microwave twice for 5 min. After washing with PBS (0.01 mmol/L, pH 7.4) thrice and each time for 5 min, sections were immersed in 3% H_2_O_2_ solution for 10 min to inactivate the endogenous peroxidase. Thereafter, 10% serum was used to block nonspecific-binding sites, and in the experimental groups, primary antibodies (the dilution ratio of primary antibodies against PTGS2 was 1 : 100) were added, respectively, onto the sections to react overnight, with nonimmune serum as negative control. Biotinylated goat anti-rabbit IgG and streptavidin-labeled horseradish peroxidase (HRP) (Proteintech Group, Inc., Rosemont, USA) were applied to react with the primary antibodies, and the DAB color solution was added to develop the immune complex as a brown precipitate. After the sections were stained with hematoxylin solution and mounted in a neutral balsam according to the general protocol, each slide was observed under light microscope (Nikon Eclipse ci, Nikon instruments company, Shanghai), and recorded by its fitted camera DS-Ri1 at 1270 pixels with a software of NIS-Elements FV.4.30.01.

### 2.7. Quantification of mRNA by RT-PCR

Total RNA was extracted using the TRIZOL reagent (Invitrogen, Carlsbad, CA, USA), followed by reverse transcription to cDNA with reverse transcriptases. Gene-specific primers were designed using Primer Express Software v2.0 (ABI, Waltham, Mass, USA). Primer sequences are shown in Table 2. The mRNA levels were detected by real-time polymerase chain reaction (PCR) (Applied Biosystems, Foster City, CA, USA) using 2 × Taq PCR Master Mix (TIANGEN, Beijing, China). All reactions were performed in triplicates, and the thermal cycling conditions were as follows: 5 min at 94°C, followed by 38 cycles at 94°C for 30 s, 57°C for 30 s, and 72°C for 30 s. *GAPDH* was used to normalize target PTGS2 mRNA levels.

### 2.8. Western Blot Analyses

Total proteins were extracted from ectopic endometrial tissues (Beyotime Biotechnology Research Institute). In each experimental group, 20 *μ*g of total protein sample was loaded per lane and subjected to 10% SDS/PAGE. The separated protein bands were transferred to polyvinylidene difluoride membranes, and the membranes were blocked for 1 h in 5% bovine serum albumin (BSA) (prepared in PBS buffer with 0.05% Tween-20) and then incubated with PTGS2 monoclonal antibodies in blocking buffer (the dilution ratio was 1 : 1,000 in 5% BSA) for 90 min at 37°C. After washing thrice with PBST, the membranes were incubated with an HRP-conjugated secondary antibodies (diluted in a ratio of 1 : 4,000 with 5% BSA) for 60 min at 37°C. The primary GAPDH antibody was diluted to 1 : 2,000 with 5% BSA and served as an internal control. The membranes were washed thrice with PBST, and photographs were taken immediately after color development using a Bio-Rad Gel Imaging System (Hercules, CA, USA). The relative levels of proteins were semiquantitatively determined using ImageJ analysis software (ImageJ2x 2.1.4.7, National Institutes of Health, Maryland, Baltimore, USA). The target protein concentration of each sample was determined as a gray value ratio relative to GAPDH.

### 2.9. Statistical Analysis

Data were presented as mean ± standard deviation. SPSS 18.0 software (Statistical Product and Service Solutions, IBM Corp., Armonk, NY, USA) was used for statistical analysis. The statistical significance among three or more groups was determined by one-way ANOVA analysis. Pairwise comparisons between group means were conducted using the least significant difference (LSD) method. Two-tailed probability at *p* < 0.05 was considered to be statistically significant.

## 3. Results

### 3.1. Effect of Chinese Medicines on the Volume and Growth Inhibitory Rates of Ectopic Endometria in an EMs Rat Model

The growth inhibition rate of the ectopic endometrium (%) was calculated as follows: (volume before treatment − volume after treatment)/volume before treatment × 100%. There was no significant difference between groups in the volume of ectopic endometrial tissue before treatment (*p* > 0.05). The volume of ectopic endometrium in each group was reduced by varying degrees after treatment. Except for the celecoxib group, the volume of ectopic endometrium after treatment in other groups was significantly lower, and the growth inhibition rate in other groups was higher than that in the model group (*p* < 0.05). Especially, the growth inhibition rate of ectopic endometrium in the Caulis Sargentodoxae prescription group was higher than that in the celecoxib and combination groups (Table 3).

### 3.2. Effects of Chinese Medicines on the Levels of Inflammation-Related Factors in the Peritoneal Fluid in Rats with EMs

After treatment, there were significant differences in the levels of IL-1, IL-2, IL-6, and TNF-*α* in the peritoneal fluid of rats in each group (*p* < 0.05). The levels of four kinds of inflammatory cytokines in the peritoneal fluid in the model group were significantly higher than that in the normal control group (*p* < 0.05). There was a pronounced decline in the levels of four kinds of inflammatory cytokines in the peritoneal fluid in all administration groups compared with the model group (*p* < 0.05). In comparison with the model group, the levels of IL-1 and IL-2 in the peritoneal fluid in each administration group evidently declined (*p* < 0.05), and they were the lowest in the combination group. The levels of IL-1 and IL-2 in the peritoneal fluid in the Caulis Sargentodoxae prescription group were lower than those in the gestrinone group. The level of IL-6 in the peritoneal fluid was the lowest in the gestrinone group and combination group. There was significant decrease in the level of IL-6 in the peritoneal fluid in all administration groups compared with the model group (*p* < 0.05). The level of TNF-*α* in the peritoneal fluid was the lowest in the ovariectomized group and combination group. There was obvious degrease in the level of TNF-*α* in the peritoneal fluid in all treatment groups compared with the model group (*p* < 0.05) (Table 4).

### 3.3. Effect of Chinese Medicines on the Level of PGE_2_ in the Serum and Peritoneal Fluid in Rats with EMs

After treatment, there was significant difference in the level of PGE_2_ in the serum and peritoneal fluid of rats in each group (*p* < 0.05). The level of PGE_2_ in the serum and peritoneal fluid in the model group was significantly higher than that in the normal control group (*p* < 0.05), and its level in the peritoneal fluid was higher than that in the serum. There was a pronounced decline in the level of PGE_2_ in the serum and peritoneal fluid in all treatment groups compared with the model group (*p* < 0.05). In comparison with the model group, the level of PGE_2_ in the serum and peritoneal fluid in the Caulis Sargentodoxae prescription group, gestrinone group, ovariectomized group, and combination group sharply decreased (*p* < 0.05) (Table 5).

### 3.4. Effects of Chinese Medicines on the Expression of PTGS2 in the Ectopic Endometrium of Rats with EMs

**Figure 1:**
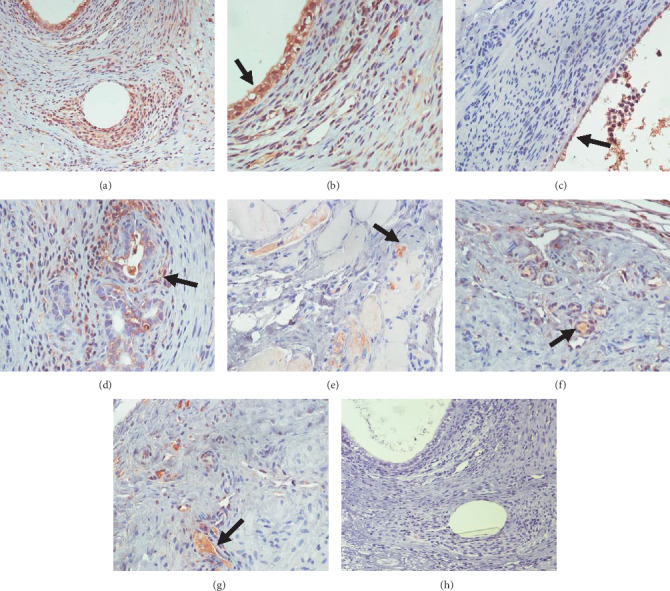
The expression of PTGS2 in ectopic endometrium tissues of rats in each group presented by IHC after DAB staining. (a) Model group, original objective magnification 20x, (b) model group, (c) ovarietomized group, (d) gestrinone group, (e) caulis Sargentodoxae prescription group, (f) celecoxib group, (g) combination group, original objective magnification 40x, and (h) negative control, original objective magnification 20x. The arrow indicates the positive site that deserves attention.

The results of the immunohistochemistry experiment (Figure 1) showed that the positive expression of PTGS2 in the ectopic endometrial tissue in the model group was strong, and which was reduced to varying degrees in all treatment groups. The positive expression of PTGS2 in the ectopic endometrium significantly decreased in the Caulis Sargentodoxae prescription group. In the gestrinone group, the expression of PTGS2 in the glandular epithelium and surrounding stromal cells was less than in the model group both in the extent and scope. The positive expression of PTGS2 in the ectopic endometrium in the celecoxib group and combination group also obviously decreased. In the ovariectomized group, the positive expression of PTGS2 was found only in the cytoplasm of the thinned luminal epithelium and some exfoliated cells in the surrounding tissues.

**Figure 2:**
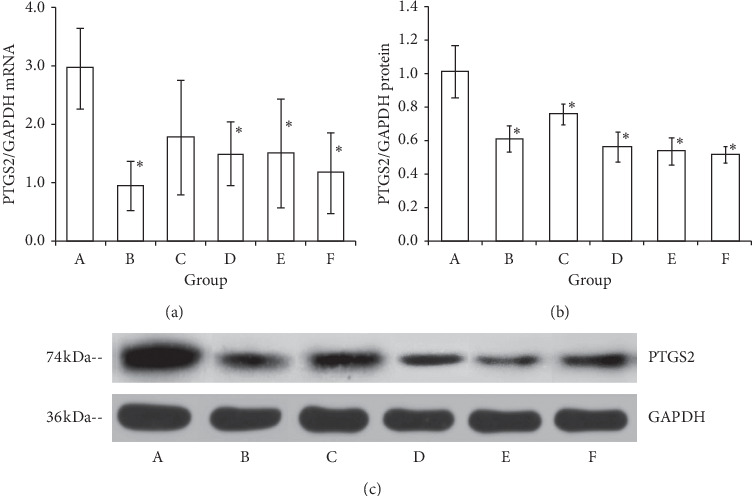
The mRNA and protein expression of PTGS2 in ectopic endometrium tissues of rats in each group. (a) The mRNA expression of PTGS2 in ectopic endometrium as assessed by real-time PCR. (b) Semiquantitative analysis on the relative levels of the protein PTGS2. (c) The photos of protein bands of PTGS2 in ectopic endometrium presented by western blotting. Lanes A–F: model group, ovariectomized group, gestrinone group, Caulis Sargentodoxae prescription group, celecoxib group, and combination group. In the bar charts, ^*∗*^*p* < 0.05, compared with the model group.

The relative expression of PTGS2 in the ectopic endometrium after treatment was assessed by real-time quantitative PCR and western blotting at mRNA and protein levels, respectively (Figure 2). When the relative protein level was semiquantitatively determined, statistical analyses were performed. The difference between groups at the mRNA level and the relative protein level by one-way variance analysis both reach a significant level (*p* < 0.05). The mRNA and protein expression of PTGS2 in the ectopic endometrium in the model group was high. The relative expression of mRNA of PTGS2 in the ectopic endometrium in the Caulis Sargentodoxae prescription group, celecoxib group, ovariectomized group, and combination group was lower than that in the model group (*p* < 0.05) (Figure 2(a)). Compared with the model group, the relative protein level of PTGS2 in the ectopic endometria in all treatment groups markedly decreased (*p* < 0.05) (Figure 2(b)).

## 4. Discussion

In this research, we focused on Caulis Sargentodoxae prescription, which consists mainly of sargent gloryvine, raw typha pollen, Rhizoma Corydalis, and peach kernel. As in the traditional Chinese medicine literature, the compatibility of various herbs plays a coordinated role in clearing heat and activating blood circulation to dissipate blood stasis. Clinical reports [14, 16–18] have shown that Caulis Sargentodoxae prescription has good curative effect on the treatment of EMs for it can obviously relieve dysmenorrhea and chronic pelvic pain, improve the clinical symptoms, and raise quality of life of EMs patients with postoperative recurrence. Furthermore, its side effects and adverse reactions are mild. Our previous results from animal experiments [19–23] showed that Caulis Sargentodoxae prescription can inhibit the growth of ectopic endometrial lesions in rats, reduce the expression of matrix metalloproteinases (MMPs) and adhesion molecules in the eutopic and ectopic endometria of rats with EMs, and decrease the hydrolysis of the extracellular matrix by the ectopic endometria. Our in vitro experiments [24] also showed that Caulis Sargentodoxae prescription can inhibit the growth of endometrial cells of rats. Although we previously performed research on Caulis Sargentodoxae prescription in the treatment of EMs, we did not recognize the inhibitory effect of Caulis Sargentodoxae prescription from dynamical and comparative perspectives, especially not its action on inflammation of EMs. In this study, the results showed that the growth inhibition rate of the ectopic endometrium was significantly higher (reaching about 59%) in the Caulis Sargentodoxae prescription group in comparison with the model group, suggesting that Caulis Sargentodoxae prescription had a significant inhibitory effect on the growth of the ectopic endometrium in rats with EMs. In addition, with the progesterone and PTGS2 receptor inhibitor as reference, the efficacy of Caulis Sargentodoxae prescription to treat EMs was further authenticated.

Since the therapeutic effects were confirmed, changes in the inflammatory microenvironment became the next issues, especially in response to PTGS2 in ectopic lesions. PGE2 can also inhibit the phagocytic function of macrophages, resulted in changes of the peritoneal microenvironment [25, 26]. Clinically, the samples commonly available for endometriosis are mainly blood and peritoneal fluid, and peritoneal fluid more directly reflects the changes of local microenvironment, while blood mainly reflects the overall endocrine and immune changes. So, in this work, inflammatory cytokines in peritoneal fluid and serum were focused. The levels of IL-1, IL-2, IL-6, and TNF-*α* in the peritoneal fluid, and the level of PGE2 in the peritoneal fluid and serum were significantly reduced in the Caulis Sargentodoxae prescription group compared to the model group, suggesting that Caulis Sargentodoxae prescription has anti-inflammatory effect on the organism level. Moreover, Caulis Sargentodoxae prescription can locally improve the inflammatory microenvironment in rats with EMs for the expression of PTGS2 in the ectopic endometrium decreased both at mRNA and protein levels. Gestrinone can reduce the level of the inflammatory cytokines, inhibit PTGS2 activity to reduce PGE2 production as Caulis Sargentodoxae prescription, and decrease PTGS2 though at mRNA level not up to significance. The PTGS2 inhibitor celecoxib can inhibit the inflammatory response in the abdominal cavity of rats by inhibiting the activity of the PTGS2 enzyme but also perhaps by reducing the expression of its gene in the ectopic endometrium. In this way, we believe that the anti-inflammatory effect of Caulis Sargentodoxae prescription can happen both at organism level and in local microenvironment, especially as a similar anti-inflammatory effect to the prostaglandin synthase (PTGS2) inhibitor (celecoxib) and progesterone antagonist (gestrinone).

PTGS2 plays a role in the physiological and pathological processes, such as pain, apoptosis, excitatory synaptic transmission, angiogenesis, and fibrosis [27, 28]. Consequently, on the inhibition of the inflammation in rats with EMs, Caulis Sargentodoxae prescription and the PTGS2 inhibitor celecoxib have both significant effects. They both can reduce the level of the inflammatory cytokines in the peritoneal cavity and the expression of PTGS2 in the ectopic endometrium, relieve the inflammatory microenvironment in the abdominal cavity of rats, and reduce the local inflammatory response. But as for gestrinone, there is similarly with an inhibitory effect on the intra-abdominal inflammatory microenvironment to downregulate the level of PGE2 in the serum and peritoneal fluid and to relieve pain, it did not reduce the expression of PTGS2 at the mRNA level in the ectopic endometrium up to a statistical significance. By comparing effects of the combination group with the Caulis Sargentodoxae prescription or celecoxib group, it was found that Caulis Sargentodoxae prescription can act as the PTGS2 inhibitor, or they have a synergistic effect. Therefore, Caulis Sargentodoxae prescription can mitigate the inflammatory microenvironment together with the PTGS2 inhibitor, and as for gestrinone, possibly in a different way despite their similar effects.

## 5. Conclusions

Therapeutic effects of Caulis Sargentodoxae prescription were verified for it reduces ectopic endometrial volumes in rats with EMs and inhibits inflammation both at the organism level and in local microenvironment. By comparing with the PTGS2 inhibitor celecoxib and progesterone antagonist (gestrinone), Caulis Sargentodoxae prescription has a similar anti-inflammation to them, suggesting possible combinative or alternative clinical application between them to treat EMs.

## Figures and Tables

**Table 1:** Different oral administrations in groups of rats.

Groups	*N*	Oral administrations	Dosage
Normal control	5	Physiological saline	10 ml/(kg·BW·d) × 21 d
Model	8	Physiological saline	10 ml/(kg·BW·d) × 21 d
Ovariectomized	8	Physiological saline	10 ml/(kg·BW·d) × 21 d
Gestrinone	8	Gestrinone	0.5 mg/(kg·BW·d) × 21 d
Caulis Sargentodoxae prescription	8	Caulis Sargentodoxae prescription	27.72 g/(kg·BW·d) × 21 d
Celecoxib	8	Celecoxib	0.025 g/(kg·BW·d) × 21 d
Combination	8	Caulis Sargentodoxae prescription + celecoxib	13.86 g/(kg·BW·d) + 0.0125 g/(kg·BW·d) × 21 d

**Table 2:** Primer sequences used for quantitative RT-PCR in this study.

Gene	Forward primer (5′-3′)	Reverse primer (5′-3′)
PTGS2	GTGCCGGGTCTGATGATGTA	TGAAGTGGTAACCGCTCAGG
GAPDH	GGCAAGTTCAACGGCACAGT	ATGACATACTCAGCACCGGC

**Table 3:** The volume and growth inhibitory rates of ectopic endometrium before and after treatment $\left(\bar{x}\pm s\right)$.

Group	*N*	Volume before treatment (mm^3^)	Volume after treatment (mm^3^)	Growth inhibitory rate (%)
Model	8	148.208 ± 40.089	103.224 ± 28.346	27.76 ± 21.95
Ovariectomized	8	149.470 ± 41.566 (*p*=0.857)	37.523 ± 7.663^*∗*^(*p*=0.001)	73.25 ± 8.35^*∗*^(*p*=0.001)
Gestrinone	7	154.425 ± 37.679 (*p*=0.499)	59.860 ± 21.167^*∗*^(*p*=0.009)	61.36 ± 8.09^*∗*^(*p*=0.003)
Caulis Sargentodoxae prescription	8	160.254 ± 37.726 (*p*=0.234)	64.944 ± 21.322^*∗*^(*p*=0.005)	58.40 ± 13.93^*∗*^(*p*=0.003)
Celecoxib	7	147.474 ± 43.941 (*p*=0.740)	89.014 ± 30.722 (*p*=0.643)	40.19 ± 8.06 (*p*=0.531)
Combination	7	150.559 ± 43.134 (*p*=0.653)	73.330 ± 29.502^*∗*^(*p*=0.039)	52.65 ± 8.66^*∗*^(*p*=0.011)
*F*	—	0.335	5.480	27.465
*p*	—	0.960	0.001	0.001

^*∗*^ *p* < 0.05, compared with the model group.

**Table 4:** The content of IL-1, IL-2, IL-6, and TNF-*α* in the peritoneal fluid after treatment $\left(\bar{x}\pm s\right)$.

Group	*N*	IL-1 (ng/L)	IL-2 (ng/L)	IL-6 (pg/ml)	TNF-*α* (ng/L)
Normal control	5	126.96 ± 15.34	993.98 ± 101.44	62.13 ± 12.42	220.80 ± 24.24
Model	8	228.80 ± 15.39☆ (*p*=0.001)	2132.55 ± 99.41☆ (*p*=0.001)	143.42 ± 7.62☆ (*p*=0.001)	511.78 ± 18.45☆ (*p*=0.001)
Ovariectomized	8	156.15 ± 32.29^*∗*^(*p*=0.001)	1365.05 ± 398.65^*∗*^(*p*=0.001)	111.38 ± 23.37^*∗*^(*p*=0.001)	263.47 ± 48.45^*∗*^(*p*=0.001)
Gestrinone	7	159.96 ± 21.22^*∗*^(*p*=0.001)	1606.71 ± 200.78^*∗*^(*p*=0.001)	95.43 ± 8.92^*∗*^(*p*=0.001)	308.22 ± 17.88^*∗*^(*p*=0.001)
Caulis Sargentodoxae prescription	8	130.30 ± 19.32^*∗*^(*p*=0.001)	1356.89 ± 279.98^*∗*^(*p*=0.001)	108.27 ± 15.45^*∗*^(*p*=0.001)	356.01 ± 47.73^*∗*^(*p*=0.001)
Celecoxib	7	147.52 ± 25.35^*∗*^(*p*=0.001)	1342.12 ± 107.68^*∗*^(*p*=0.001)	111.25 ± 19.78^*∗*^(*p*=0.001)	309.57 ± 38.81^*∗*^(*p*=0.001)
Combination	7	128.75 ± 24.85^*∗*^(*p*=0.001)	1168.52 ± 194.83^*∗*^(*p*=0.001)	91.43 ± 15.70^*∗*^(*p*=0.001)	274.11 ± 31.82^*∗*^(*p*=0.001)
*F*	—	8.529	8.118	9.102	28.595
*p*	—	0.001	<0.001	0.001	<0.001

^*∗*^ *p* < 0.05, compared with the model group. ☆ expresses *p* < 0.05, compared with the normal control group.

**Table 5:** The content of PGE_2_ in the serum and peritoneal fluid $\left(\bar{x}\pm s\right)$.

Group	*N*	PGE_2_ (ng/L)	
		Serum	Peritoneal fluid
Normal control	5	240.95 ± 25.13	260.01 ± 27.28
Model	8	478.39 ± 77.40☆ (*p*=0.001)	505.20 ± 25.83☆ (*p*=0.001)
Ovariectomized	8	313.27 ± 49.15^*∗*^(*p*=0.001)	371.30 ± 36.57^*∗*^(*p*=0.001)
Gestrinone	7	303.94 ± 46.61^*∗*^(*p*=0.001)	334.92 ± 29.18^*∗*^(*p*=0.001)
Caulis Sargentodoxae prescription	8	365.71 ± 102.61^*∗*^(*p*=0.001)	336.21 ± 75.96^*∗*^(*p*=0.001)
Celecoxib	7	413.80 ± 79.68 (*p*=0.075)	373.57 ± 58.15^*∗*^(*p*=0.001)
Combination	7	342.31 ± 56.21^*∗*^(*p*=0.001)	336.22 ± 53.67^*∗*^(*p*=0.001)
*F*	—	11.656	8.104
*p*	—	0.001	0.001

^*∗*^ *p* < 0.05, compared with the model group. ☆ indicates *p* < 0.05, compared with the normal control group.

## Data Availability

The data used to support the findings of this study are available from the corresponding author upon request.

## Abbreviations

EMs: Endometriosis
TCM: Traditional Chinese medicine
PTGS2: Prostaglandin endoperoxide synthase 2
IL: Interleukin
TNF-*α*: Tumor necrosis factor-*α*
PGE2: Prostaglandin E2
MCP-1: Monocyte chemotactic protein 1
SIPPR: Shanghai Institute of Planned Parenthood Research
API: Active pharmaceutical ingredient
GAPDH: Glyceraldehyde-3-phosphate dehydrogenase
PBS: Phosphate-buffered saline
HRP: Horseradish peroxidase
PCR: Polymerase chain reaction
BSA: Bovine serum albumin
LSD: Least significant difference
MMPs: Matrix metalloproteinases.

## References

[1] Xie X., Gou W. (2013). *Gynecotokology*.

[2] Giudice L. C. (2010). Endometriosis. *New England Journal of Medicine*.

[3] McLeod B. S., Retzloff M. G. (2010). Epidemiology of endometriosis: an assessment of risk factors. *Clinical Obstetrics and Gynecology*.

[4] Selcuk I., Bozdag G. (2013). Recurrence of endometriosis; risk factors, mechanisms and biomarkers; review of the literature. *Journal of the Turkish German Gynecological Association*.

[5] Group CMAoaGbec (2015). Guidelines for the diagnosis and treatment of endometriosis. *Chinese Journal of Obstetrics and Gynecology*.

[6] Sharpe-Timms K. L., Zimmer R. L., Ricke E. A., Piva M., Horowitz G. M. (2002). Endometriotic haptoglobin binds to peritoneal macrophages and alters their function in women with endometriosis. *Fertility and Sterility*.

[7] Capobianco A., Rovere-Querini P. (2013). Endometriosis, a disease of the macrophage. *Frontiers in Immunology*.

[8] Cao X., Yang D., Song M., Murphy A., Parthasarathy S. (2004). The presence of endometrial cells in the peritoneal cavity enhances monocyte recruitment and induces inflammatory cytokines in mice: implications for endometriosis. *Fertility and Sterility*.

[9] Shu L., Song Z., Dai D. (1999). Clinical observation of 80 cases of sargentgloryvine mixture in the treatment of endometriosis. *Journal of Traditional Chinese Medicine*.

[10] Shu L. (1999). Clinical observation of sargentgloryvine oral liquid in the treatment on endometriosis. *Shanghai Journal of Traditional Chinese Medicine*.

[11] Song Z., Shu L., Dai D. (2000). The clinical observation on removing heat and activating stasis in the treatment of endometriosis. *Hubei Journal of Traditional Chinese Medicine*.

[12] Yin Y., Zhang T., Huang C., Shu L. (2007). 31 cases of caulis sargentodoxae treatment on recurrent endometriosis after operation. *Journal of Practical Traditional Chinese Medicine*.

[13] Yin Y., Cao Y., Zhang T. (2011). Efficacy and life quality assessment of 41 cases of recurrent endometriosis treated by removing heat and activating stasis methods after operation. *Journal of Shanghai University of Traditional Chinese Medicine*.

[14] Yang F., Li F., Shu L., Zhang T. (2006). Clinical observation of 82 cases of caulis sargentodoxae prescription in the treatment of endometriosis. *Shandong Journal of Traditional Chinese Medicine*.

[15] Jones R. C. (1987). The effect of a luteinizing hormone-releasing hormone antagonist on experimental endometriosis in the rat. *Acta Endocrinologica*.

[16] Shu L., Zhang T., Chen J., Shen M. (2010). Application of caulis sargentodoxae prescription in the treatment of gynecological pain. *Journal of Chinese Modern Traditional Chinese Medicine*.

[17] Yin Y., Cao Y., Zhang T. (2011). Effects of Hongteng recipe on postoperative recurrent endometriosis and quality of life: a report of 41 cases. *Journal of Shanghai University of Traditional Chinese Medicine*.

[18] Cao Y., Zhao L., Chen H., Xu M. (2013). Professor Dai deying’s experience in treating endometriosis with “hongteng prescription. *SH Journal of Traditional Chinese Medicine*.

[19] Zhang T., Liu J., Shu L., Dai D. (2005). Interference of compound caulis sargentodoxae granule in expression of matrix metalloproteinase2 and matrix metalloproteinase9 in rats with endometriosis. *Journal of Shanghai University of Traditional Chinese Medicine*.

[20] Zhang T., Chen Q., Zhu K., Cao L. (2005). The drug effects of traditional Chinese medicine “sargentgloryyine formula” on experimental endometriosis in the rat. *Reproduction & Contraception*.

[21] Zhang T., Qin B., Cao Y., Xie S. (2008). Effect of “caulis sargentodoxae formula” on aromatase expression in endometrial rats. *Reproduction & Contraception*.

[22] Cao Y., Zhang T., Xie S., Zhu Y. (2009). Effects of caulis sargentodoxae granule on expressions of vascular endothelial growth factor and its receptor-2 in rats with endometriosis. *Journal of Chinese Integrative Medicine*.

[23] Cao Y., Zhu Y., Qin B., Xie S. (2011). Effect of traditional Chinese medicine “caulis sargentodoxae drug granuleson” on gene expression in adhesive molecule and VEGFmRNA of ectopic endometrium in endometriosis model rats. *Reproduction & Contraception*.

[24] Liu J., Zhang T., Shu L., Dai D. (2009). Effect of caulis sargentodoxae prescription on the growth of endometrial cells cultured in vitro in rats. *Tianjin Journal of Traditional Chinese Medicine*.

[25] Chuang P. C., Lin Y. J., Wu M. H., Wing L.-Y. C., Shoji Y., Tsai S.-J. (2010). Inhibition of CD36-dependent phagocytosis by prostaglandin E2 contributes to the development of endometriosis. *The American Journal of Pathology*.

[26] Wu M. H., Chuang P. C., Lin Y. J., Tsai S. J. (2013). Suppression of annexin A2 by prostaglandin E2 impairs phagocytic ability of peritoneal macrophages in women with endometriosis. *Human Reproduction*.

[27] Frungieri M. B., Weidinger S., Meineke V., Kohn F. M., Mayerhofer A. (2002). Proliferative action of mast-cell tryptase is mediated by PAR2, COX2, prostaglandins, and PPARgamma: possible relevance to human fibrotic disorders. *Proceedings of the National Academy of Sciences of USA*.

[28] Bauman K. A., Wettlaufer S. H., Okunishi K. (2010). The antifibrotic effects of plasminogen activation occur via prostaglandin E2 synthesis in humans and mice. *Journal of Clinical Investigation*.

